# Yeast Models of Prion-Like Proteins That Cause Amyotrophic Lateral Sclerosis Reveal Pathogenic Mechanisms

**DOI:** 10.3389/fnmol.2018.00453

**Published:** 2018-12-11

**Authors:** Zachary T. Monahan, Shannon N. Rhoads, Debra S. Yee, Frank P. Shewmaker

**Affiliations:** Department of Pharmacology and Molecular Therapeutics, Uniformed Services University, Bethesda, MD, United States

**Keywords:** prion, FUS, TAF15, HNRNPA2B1, EWSR1, TDP-43, amyloid, ALS

## Abstract

Many proteins involved in the pathogenic mechanisms of amyotrophic lateral sclerosis (ALS) are remarkably similar to proteins that form prions in the yeast *Saccharomyces cerevisiae*. These ALS-associated proteins are not orthologs of yeast prion proteins, but are similar in having long, intrinsically disordered domains that are rich in hydrophilic amino acids. These so-called prion-like domains are particularly aggregation-prone and are hypothesized to participate in the mislocalization and misfolding processes that occur in the motor neurons of ALS patients. Methods developed for characterizing yeast prions have been adapted to studying ALS-linked proteins containing prion-like domains. These yeast models have yielded major discoveries, including identification of new ALS genetic risk factors, new ALS-causing gene mutations and insights into how disease mutations enhance protein aggregation.

## Introduction

Neurodegenerative diseases are defined by irreversible loss of neurons. Clinically, these diseases manifest as dementias and movement disorders, depending on the subset of neurons that are principally affected. As a whole, the cost of neurodegenerative diseases is staggering. According to the Alzheimer’s Association, in 2018, the total expense of treating dementias in the United States was approximately $277 billion. Unlike cancer and viral diseases, which have seen astonishing advances in treatments, no drugs have been developed that reverse or stop progression of neurodegenerative disease. To develop effective treatments, there is need to better understand the fundamental causes of these diseases and develop tractable models for genetic and pharmacological screening.

Amyotrophic lateral sclerosis (ALS) is a neurodegenerative disease that affects upper and lower motor neurons. It primarily manifests as a movement disorder, but it has many clinical and pathological features that overlap with frontotemporal dementia (FTD), which primarily manifests as a dementia. A surprising development in recent years is that diseased neurons from ALS and FTD patients frequently feature the accumulation of specific proteins that resemble yeast prion proteins. The intersection of yeast prions and human disease-associated proteins has led to novel experimental systems using tractable yeast models.

### Prions

A common theme among nearly all neurodegenerative diseases is an apparent loss of normal protein quality control, resulting in the accumulation of disease-specific proteins into large aggregates. Increasingly, a prion-like mechanism is being recognized as potentially underlying this protein aggregation (Polymenidou and Cleveland, 2011). The term “prion” means infectious protein (Table 1). However, infectivity does not necessarily result from the protein itself, but from its folded tertiary and quaternary structure. Mechanistically, a prion protein adopts a multimeric, highly-ordered conformation, which can then propagate through healthy cells and tissue by causing similar proteins to adopt the pathological form. This is distinct from protein misfolding into amorphous aggregates within isolated cells because prion propagation is a molecular pathological process that can spread. The accumulation of various proteins into large aggregates are hypothesized to cause neuronal degeneration by causing loss of the proteins’ normal functions, as well as causing gain-of-function toxic properties.

**Table 1:** Concepts linking yeast prions and subtypes of amyotrophic lateral sclerosis (ALS) and frontotemporal dementia (FTD).

Amyloid	A structurally ordered filamentous aggregate consisting of a single protein in a polymeric arrangement; each end of the filament serves as template for the addition of more protein, providing a molecular mechanism for runaway aggregation. Several ALS-linked proteins can form amyloid with atypical dye-binding properties.
Neuronal cytoplasmic inclusions (NCIs)	Large pathological protein aggregates that commonly form in neurons afflicted by various neurodegenerative diseases. Different diseases frequently have a unique subset of proteins within NCIs. Similar inclusions are observed in yeast cells harboring specific prion proteins.
Prion	An infectious protein. Canonically, a prion protein adopts an alternative structural fold that causes similar protein molecules to adopt the same structure (e.g., amyloid). Most characterized prion proteins are found in fungi, particularly *Saccharomyces cerevisiae*.
Prion domain	The distinct segments of yeast proteins that enable prion formation. These domains are distinguished by their “low-complexity,” consisting predominately of hydrophilic, but not charged or hydrophobic, amino acids.
Prion-like domain	Segments found in many mammalian proteins, especially proteins with RNA-related functions, which have sequence composition very similar to yeast prion domains. They are disproportionately present in proteins that aggregate in neurodegenerative diseases.

For years, infectious forms of the mammalian prion protein, or PrP, were the only protein-only agents thought to be responsible for a human disease (Prusiner, 2013). Collectively, these diseases are known as the transmissible spongiform encephalopathies. The idea that a protein could itself be an infectious agent was initially controversial. However, the protein-only model of infectivity gained significant support from experiments demonstrating the ability to generate infectious prions *in vitro*. Castilla et al. (2005) generated infectious PrP using an *in vitro* amplification protocol from infected brain material. Later work strengthened the protein-only infectivity model by producing prions from bacteria-derived recombinant PrP (Wang et al., 2010; Deleault et al., 2012).

The discovery that the yeast *Saccharomyces cerevisiae*—one of the most widely used eukaryotic model systems—harbored naturally occurring prions completely unrelated to PrP significantly increased the acceptance of the prion hypothesis (Wickner, 1994). In the years since discovery of the first yeast prions (e.g., Rnq1p, Sup35p, Ure2p), dozens of yeast proteins have been proposed to have “prion properties,” which broadly means the proteins can stably (but reversibly) exist within a cell population in one of two forms: (1) a soluble and functional form; or (2) a dysfunctional and high-molecular-weight aggregated form, concentrated mostly within discrete, insoluble, punctate ultrastructures. For example, an isogenic yeast strain might be cultured as two distinct populations. In one population a specific prion protein is aggregated within all cells, while in the other population the protein is soluble in all cells. Unlike PrP, yeast prions are generally tolerated by host cells, causing only mild phenotypic effects, which make them tractable experimental models for studying the prion phenomenon. The phenotypes resulting from yeast prions are generally associated with diminished function of the specific prion protein because it is accumulated into aggregates.

### Amyloid and Prion Domains

The ability of yeast prion proteins to adopt an alternative, aggregated state is a result of their ability to fold into a multimeric structural motif known as amyloid (Table 1). Amyloid is a filamentous protein homopolymer of indefinite length in which the protein subunits stack linearly to form beta sheets that run the length of the polymer. Individual beta strands run perpendicular to the filament’s long axis. The amyloid formed *in vitro* by purified recombinantly produced yeast prion proteins is infectious to yeast, thus proving the amyloid/protein-only model of yeast prions (King and Diaz-Avalos, 2004; Tanaka et al., 2004; Brachmann et al., 2005; Patel and Liebman, 2007).

Importantly, amyloid is a well-ordered, folded state, conceptually like a one-dimensional crystal. The significance of this architecture is it tends to be relatively stable, thus resistant to treatments that might normally unfold or solubilize protein aggregates, and it provides a mechanism for self-propagation. The ends of the filaments serve as templates for the autocatalytic conversion of additional protein into the amyloid conformation. The breaking of filaments provides new sites for protein recruitment. Moreover, amyloid filaments of short length (or oligomeric amyloid) can remain relatively soluble and be passed to progeny yeast cells. In fact, for several neurodegenerative diseases, smaller oligomeric assemblies, which are pre-amyloid or amyloid-like, may in fact be the more cytotoxic and infectious species (Espargaró et al., 2016).

In the case of disease, the concern with amyloid is that it has the potential to cause runaway aggregation of a specific protein, while resisting the cellular mechanisms dedicated to clearing misfolded proteins. Also, as with crystal structures, a single protein can adopt multiple slightly different amyloid conformations that differ at the atomic level, and perhaps, disease presentation. These are called variants, or strains, and can differ in their biological effects (Prusiner, 1998; Diaz-Avalos et al., 2005). It is important to note that there could be other higher-order filamentous pathogenic structures that differ from canonical amyloid but are likewise able to propagate through template-driven growth.

Most yeast prion proteins assemble into amyloid via their aptly named “prion domains.” These are lengthy, intrinsically disordered regions composed disproportionately of just a few amino acids (i.e., low-complexity). They are distinguished by an abundance of polar residues and a paucity of hydrophobic and charged residues (Toombs et al., 2010). Their removal from a prion protein eliminates its ability to be a prion. Numerous human proteins have segments that highly resemble yeast prion domains; they are generally called “prion-like” because it is unknown if they have any prion-facilitating properties *in vivo* (Table 1). In recent years, several human proteins with prion-like domains have been implicated in neurodegenerative diseases. Importantly, human prion-like proteins are extremely over-represented in genetic links to neurodegenerative disease (An and Harrison, 2016). Strikingly, many of these prion-like proteins are found in the large pathological aggregates of diseased neurons. In general, prion-like domains facilitate self-assembly into larger complexes and aggregates (March et al., 2016). For example, prion-like domains can be exchanged for true yeast prion domains with a retention of capacity for functional aggregation (Shelkovnikova et al., 2014). This has led to speculation that some of these proteins harboring prion-like domains may contribute to pathology via a mechanism similar to what is observed with naturally occurring yeast prions.

This emerging model suggests that autocatalytic protein propagation, the fundamental phenomenon of prion spread, potentially underlies a much broader collection of neurodegenerative diseases than just the previously described transmissible spongiform encephalopathies. The specific contribution of a prion mechanism to diseases like ALS, Parkinson’s, Alzheimer’s and chronic traumatic encephalopathy remains controversial, but there is ample evidence supporting the possibility. For example, aggregates of neurodegenerative disease-linked proteins can “infect” cell models causing endogenous proteins to aggregate (Furukawa et al., 2011; Karpowicz et al., 2017; Olsson et al., 2018). Likewise, ALS-linked proteins are capable of being transferred between cultured cells (Feiler et al., 2015; Feuillette et al., 2017). Likewise, the yeast protein Sup35 can propagate as a prion via horizontal transfer between cultured mammalian cells (Krammer et al., 2009; Liu et al., 2017). All of these observations suggest infectious protein aggregates might travel anatomical pathways and cause disease.

### Why Use Yeast Models for Human Disease Proteins?

Approximately 6,000 human genes have yeast homologs; about 10% of which can be complemented by (or can complement) their yeast counterpart *in vivo* (Cherry et al., 2012). About 500 human disease genes have yeast orthologs (Kryndushkin and Shewmaker, 2011). The most straightforward approach to looking at human disease genes in yeast is in situations when the gene complements a yeast ortholog. This is the case for SOD1, in which human mutants can be quickly assayed for their ability to complement the yeast ortholog (Kryndushkin and Shewmaker, 2011). Unfortunately, most neurodegenerative disease-causing proteins do not have yeast orthologs, thus making their study more challenging. However, the methodology for studying the aggregation and propagation of yeast prion domains within genetically optimized yeast reporter strains has been refined for over two decades. These yeast models have proven efficient and inexpensive platforms for studying the potential prion-like properties of several neurodegenerative disease-linked proteins. The following sections summarize the modeling of specific ALS-associated prion-like proteins in yeast models.

## Examples of ALS-Linked Proteins With Prion-Like Domains Modeled in Yeast

**Figure 1:**
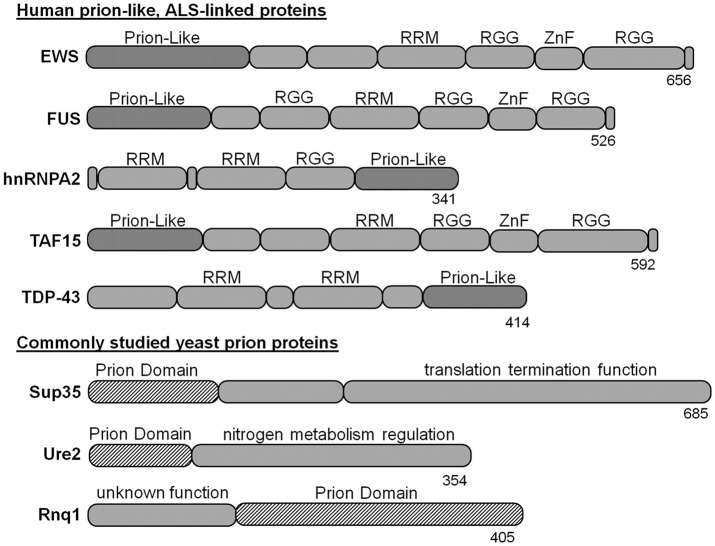
Amyotrophic lateral sclerosis (ALS)-linked proteins with yeast prion-like domains have been modeled in yeast. The prion domains of naturally occurring yeast prion proteins have similar size and composition, but are not homologous to the prion-like domains of ALS-linked proteins. The tools for studying yeast prions have been applied to ectopic expression of human disease proteins to evaluate aggregation and toxicity mechanisms. RRM, RNA recognition motif; RGG, arginine-glycine-glycine rich domain; ZnF, zinc finger domain.

Many neurodegenerative disease-linked proteins have been modeled in yeast, including Parkinson’s, Huntington’s and Alzheimer’s diseases (Braun, 2015). Likewise, there are yeast models for evaluating ALS-linked proteins, such as SOD1 and OPTN (Rabizadeh et al., 1995; Kryndushkin et al., 2012). However, yeast models of ALS-associated proteins with prion-like domains have proven particularly powerful because of their similarity to naturally occurring yeast prions. Prominent examples include the proteins fused-in-sarcoma (FUS), TAR DNA-binding protein-43 kDa (TDP-43kDa), heterogeneous nuclear ribonucleoprotein (hnRNPA2), TATA-box binding protein associated factor 15 (TAF15) and ewing sarcoma breakpoint region 1 (EWS; Figure 1), which are unified by remarkable similarities in architecture. Also, each is found post-mortem within neuronal cytoplasmic inclusions of subsets of patients with ALS or FTD. In cell models and yeast, these proteins (or their mutant forms) generally have dominant gain-of-function toxicity that is at least partially mediated by aggregation via their prion-like domains.

### FUS

FUS is a ubiquitously expressed, predominantly nuclear animal protein originally named for its association to liposarcomas (Pérez-Losada et al., 2000). In the past decade, much research on FUS has focused on its relationship to ALS and FTD (Kwiatkowski et al., 2009; Vance et al., 2009). Mutations in FUS cause around 5% of all familial ALS cases, with disease phenotypes inherited in an autosomal dominant fashion (Shang and Huang, 2016). Yeast do not encode a FUS ortholog. Yeast models that ectopically express human FUS (or disease-causing mutants) reveal gain-of-function toxicity that is tightly associated with the degree to which FUS forms aggregates in the yeast cytoplasm (Kryndushkin et al., 2012; Monahan et al., 2017). Aggregation is dependent on the presence of FUS’s prion-like domain; truncations lacking the domain are not toxic and remain relatively soluble in yeast cytoplasm (Ju et al., 2011; Kryndushkin et al., 2011; Sun et al., 2011). An important distinction between ectopic FUS and yeast prions like Sup35 is that FUS does not appear to exist as either soluble or aggregated (Kryndushkin et al., 2011), but instead appears to be sufficiently aggregation-prone in yeast cytoplasm that it always forms aggregates.

### TDP-43

Like FUS, TDP-43 is an RNA-binding protein associated with several RNA-homeostatic functions. It is mostly localized within the nucleus, but is found in neuronal cytoplasmic inclusions of FTD and ALS patients (Neumann et al., 2006). Most ALS patients have TDP-43 pathology in spinal neurons despite having no mutations in the gene (Mackenzie et al., 2007); however, mutations in TDP-43 are also responsible for a small percentage of ALS (Sreedharan et al., 2008). TDP-43’s propensity to aggregate and cause toxicity is well established in yeast models (Johnson et al., 2008; Braun et al., 2011). Yeast do not have a TDP-43 ortholog; ectopic expression of human TDP-43 indicates its carboxy-terminal prion-like domain is critical to both intracellular aggregation and toxicity (Johnson et al., 2008).

### hnRNPA2

hnRNPs function in the processing, metabolism and transport of mRNA. Yeast have several proteins with similar functions and domain architecture as human hnRNPs. Two human paralogs have been linked to ALS: hnRNPA2/B1 and hnRNPA1 (Kim et al., 2013). hnRNPA2/B1 can be alternatively spliced into A2 or B1 isoforms; A2 is the major isoform. Recombinant forms of hnRNPA1 and hnRNPA2 form filamentous structures that can self-propagate *in vitro*, and hnRNPA2 has been found in neuronal cytoplasmic inclusions (Kim et al., 2013). hnRNPA2 has a carboxy-terminal prion-like domain that has been extensively modeled in yeast for its intrinsic aggregation propensity. In yeast, known disease-causing mutations enhance prion-like aggregation relative to wild-type protein (Paul et al., 2017; Cascarina et al., 2018).

### TAF15

TAF15 is similar to FUS in both primary sequence and domain organization. Likewise, it possesses an amino-terminal prion-like domain. TAF15 is a member of the RNA polymerase II initiation complex, where it has a transcriptional regulatory role. Like FUS, there is no definitive yeast paralog of TAF15. Missense mutations in TAF15 are linked to a very small percentage of ALS patients (Couthouis et al., 2011). These mutations cause TAF15 to accumulate in the cytoplasm of neuronal models (Couthouis et al., 2011). Similarly, when human TAF15 is expressed ectopically in yeast it forms numerous cytoplasmic aggregates and causes mild cytotoxicity (Couthouis et al., 2011; Jackrel and Shorter, 2014).

### EWSR1

The EWSR1 gene codes for EWS, a protein similar to both FUS and TAF15 in domain organization. Like FUS, it gets its name from being originally identified in connection to certain types of sarcoma. EWS contains an amino-terminal prion-like domain. Modeling in yeast by Couthouis et al. (2012) revealed EWS is aggregation-prone and toxic when over-expressed, albeit to a lesser extent than TDP-43 and FUS. They also found EWS accumulated in the cytoplasm of spinal cord neurons of patients with ALS. When they sequenced carboxy-terminal exons of EWSR1, two patient-specific mutations were identified that were absent from controls.

## The Tools Developed for Yeast Prions Have Yielded High-Impact Discoveries About ALS-Linked Prion-Like Proteins

Experimentation with yeast prion proteins has largely focused on their ability to stably exist in distinct biophysical states and how interaction with chaperones and other proteins affects their aggregation, propagation and toxicity. ALS-linked proteins in yeast do not generally appear to exist as completely un-aggregated, like true yeast prions can. However, there are interactions with similar sets of chaperones, such as Hsp104 and Hsp40s, which govern how both types of proteins aggregate. These same approaches are adaptable to the study of human prion-like proteins that cause ALS.

### Evaluating the Aggregation and Toxicity Potential of Disease-Causing Mutations

Since FUS, TDP-43, hnRNPA2, TAF15 and EWS each form cytoplasmic aggregates when ectopically expressed in yeast—much like naturally occurring yeast prion proteins—it has been straightforward to monitor how specific ALS-linked mutations alter the proteins’ intrinsic propensity to aggregate and exert toxicity, especially when coupled with *in vitro* aggregation assays.

#### FUS

Most ALS-causing mutations in FUS cluster in and near its carboxy-terminal nuclear localization signal (NLS; Dormann et al., 2010). In yeast models, FUS’s NLS is insufficient to achieve dramatic nuclear localization (Ju et al., 2011). For this reason, wild-type human FUS can accumulate in the cytoplasm and cause toxicity. Using a yeast model, Sun et al. (2011) found that increasing the potency of FUS’s NLS concomitantly reduced cytoplasmic aggregation and cytotoxicity, supporting the idea that increasing nuclear localization offers a therapeutic mechanism. Likewise, the R524S and P525L ALS mutations at the carboxy terminus caused FUS to have slightly greater cytotoxicity in a yeast model (Fushimi et al., 2011), perhaps due to further diminished nuclear localization. However, another study found ALS FUS mutations to not dramatically affect aggregation and toxicity in the yeast model (Sun et al., 2011). Such a negative result is potentially informative. In this case, it suggests that the toxic mechanism of FUS aggregates has at least a component of gain-of-cytoplasmic-function, and FUS-linked toxicity is unlikely to be explained entirely by loss of function elsewhere in the cell (Sharma et al., 2016).

#### TDP-43

Most ALS-linked mutations in TDP-43 are located in its prion-like domain (Da Cruz and Cleveland, 2011). Mutant TDP-43 tested in yeast revealed that many mutations simultaneously increased aggregation propensity and toxicity (Johnson et al., 2009). However, some mutations did not accelerate TDP-43 misfolding or enhance its toxicity in yeast, which suggests mutations do not necessarily have to alter aggregation propensity to exert pathogenic effects. TDP-43 aggregates were mildly detergent resistant, but did not have the same amyloid characteristics typical of yeast prions (Johnson et al., 2009).

#### hnRNPA2

Kim et al. (2013) discovered that a single amino acid substitution (D to V) in the prion-like domain of hnRNPA2 was genetically linked to multisystem proteinopathy, an ALS-related disorder. They substituted the core of this prion-like region into the prion-nucleating segment of the yeast prion protein Sup35 (known as [PSI^+^] in the prion form; discussed more below). In this context, they found the D to V mutation dramatically and significantly increased prion formation propensity. Thus, not only could this mutated hnRNPA2 sequence promote Sup35’s nucleation into a prion, the D to V mutation specifically facilitates prion-like aggregation.

In summary, yeast models reveal that ALS-causing mutations in prion-like proteins often increase the proteins’ intrinsic propensity to form solid cytoplasmic aggregates and/or nucleate aggregation. The advantage to using yeast models is scores of mutations can be assayed rapidly before more expensive model systems are selected for further analysis. High throughput genomic sequencing has revealed numerous low-frequency polymorphisms in ALS-linked proteins, thus yeast could be helpful in determining if certain mutations actually contribute to disease.

### Evaluating Effects of Post-translational Modifications on Aggregation and Toxicity

**Figure 2:**
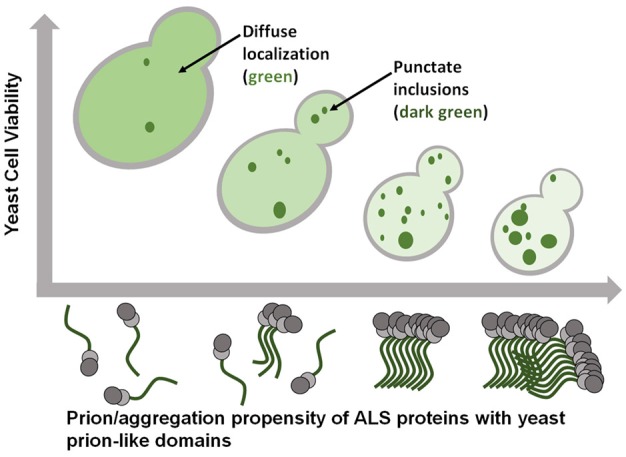
ALS-linked proteins with prion-like domains exhibit gain-of-function toxicity that is tightly associated with the extent of their aggregation. Smaller, more soluble, oligomeric aggregates may be the most toxic species.

The prion-like domain of FUS is extensively phosphorylated in human cell models following various types of stress (Rhoads et al., 2018a,b). There are as many as 12 PIK kinase sites that are phosphorylated following DNA damage. The generation of phosphomimetic substitutions within FUS’s prion-like domain have been used to model how phosphorylation could alter the behavior of FUS in a crowded cellular environment (Monahan et al., 2017). From 1 to 12 glutamate substitutions were introduced in place of the serine/threonine phosphorylation sites in the prion-like domain. These phosphomimetic substitutions caused a reduction in toxicity and aggregation in proportion to the number of substitutions, without affecting FUS expression levels. Thus incremental reduction in FUS aggregation led to incremental reduction in FUS toxicity (Figure 2). The very precise relationship between these two parameters strongly suggests that FUS aggregation is in fact causally related to cytotoxicity. Additionally, these results are consistent with similar findings reported for both alpha-synuclein in yeast (Tenreiro et al., 2014) and TDP-43 in mammalian cell models (Li et al., 2011).

Excitingly, these results offer context for observations made in post-mortem human tissue interrogated with immunohistochemistry. One critical observation has been the hyperphosphorylated state of disease-associated protein inclusions in tissue from patients with neurodegeneration (Arai et al., 2010), leading to a prevailing model wherein phosphorylation promotes protein aggregation. The use of phosphomimetics in yeast models therefore suggests that the relationship between the phosphorylation and accumulation of pathological proteins may be more nuanced than previously appreciated. If FUS is forming an archetypal amyloid structure with in-register beta sheet, as determined in the lab of Robert Tycko (Murray et al., 2017) then charged groups in the prion-like domain should strongly inhibit amyloid formation.

### Quantitative Mutagenesis to Score Prion Propensity of Human Disease Proteins

In recent years, several predictive algorithms (PAPAs) have been developed to identify proteins with yeast prion-like domains (Alberti et al., 2009; Ross et al., 2013; Lancaster et al., 2014). Largely based on their sequence composition, several 100 human proteins with potential prion capability have been identified. As a group, these proteins are very disproportionately linked to neurodegenerative disease (An and Harrison, 2016). FUS, TDP-43, hnRNPA2, TAF15 and EWS are all predicted to have prion-like behavior to various degrees. However, algorithms designed to identify large domains of particular composition may lack the resolution required to determine the effects of single amino acid substitutions.

**Figure 3:**
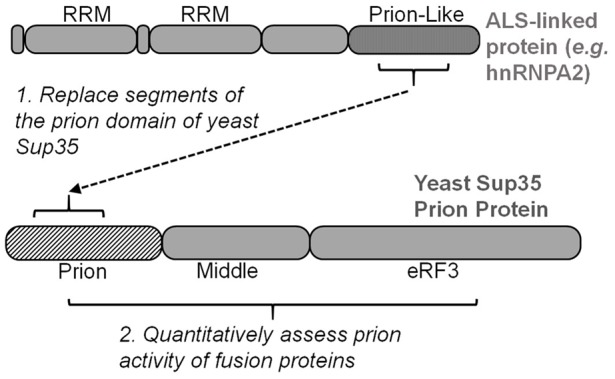
Many yeast strains have been optimized for evaluating and scoring the yeast prion protein Sup35. Inserting segments of ALS-linked prion-like domains into Sup35’s prion domain enable quantitative mutagenesis experiments to score prion propensity of amino acid mutations and substitutions. eRF3, eukaryotic release factor 3.

Simple experiments that detect the yeast prion [PSI^+^] (prion form of Sup35) can be harnessed to evaluate the nucleation potential of human prion-like proteins. Specifically, prion-like domain sequences are substituted for segments within the Sup35 prion domain (Alberti et al., 2009; Figure 3). These fusion proteins can be scored for their prion behavior using the same tools that were developed for characterizing [PSI^+^], which have sufficient resolution for quantitative analysis of specific mutations. Similar to testing how disease-linked mutations can affect aggregation and toxicity of the full-length ALS proteins, site-directed mutagenesis of the fusion protein can be used to determine how any substitution may specifically affect prion-like protein behavior.

This approach has been extensively performed with hnRNPA1 and hnRNPA2 in the lab of Eric Ross (Cascarina et al., 2018). The intrinsic aggregation propensity of hnRNPA2 was predicted* in silico* and then quantified in a yeast model (Paul et al., 2017). The central region of mutant hnRNPA2’s prion-like domain can support prion function of Sup35 when swapped with a segment of Sup35’s prion domain (residues 3–40; Figure 3; Kim et al., 2013). This hnRNPA2-Sup35 fusion protein was used to quantify how specific amino acid changes affect the prion-like aggregation of hnRNPA2 (Paul et al., 2017). Using this reporter system in combination with a PAPA, Paul et al. (2017) were able to accurately predict the prion effect of nearly every amino acid substitution—disease causing or not—within hnRNPA2’s core prion domain. Hydrophobic and aromatic residues promoted aggregation, while charged amino acids were inhibitory (Paul et al., 2017).

The hnRNPA2 yeast model also provided an important conceptual point: the hnRNPA2-Sup35 fusion proteins formed canonical prions, which means they could either remain soluble or form self-propagating amyloid (Cascarina et al., 2018). This supports the idea that these human prion-like proteins may similarly form pathological amyloid in diseased neurons. This could therefore explain why the previously mentioned hnRNPA2 D290V mutation causes disease; the loss of a charged residue in a critical amyloid-forming domain fails to inhibit amyloid formation. As new disease-linked mutations are discovered, *in silico* prediction can be coupled with rapid-throughput quantitative yeast prion models to predict amino acid effects on pathogenic aggregation.

Another discovery provided by yeast modeling is that effects of amino-acid substitutions within hnRNPA2’s core prion-like domain can indirectly alter aggregation propensity. Cascarina and coworkers discovered that the Q/N content of the core prion-like domain had important effects on proteosome-dependent clearance. While the addition of aromatic amino acids to hnRNPA2’s core prion-like domain directly increased aggregation propensity, adding Q/N increased prion formation and aggregation stochastically by inhibiting proteosome turnover (Cascarina et al., 2018).

### High-Throughput Screens

Yeast models are powerful for screening because whole-genome libraries can be quickly and inexpensively screened and analyzed. For neurodegenerative disease yeast models, libraries have been screened for genes that alter the toxicity resulting from expression/aggregation of specific disease-causing proteins (Braun et al., 2010). Screens are generally performed with knock down or over-expression libraries to observe changes in protein-induced toxicity. Importantly, the mechanisms of toxicity are unique to the different disease-causing proteins (Figley and Gitler, 2013).

Two large-scale screens of genetic modifiers of FUS toxicity yielded a consistent set of DNA/RNA-binding proteins (Ju et al., 2011; Sun et al., 2011). This corroborates observations that interactions with RNA are integral to FUS-induced toxicity. A yeast RNA helicase, ECM32, was discovered in both screens to suppress FUS-induced toxicity (Daigle et al., 2013). Its human homolog, hUPF1, was likewise found to suppress FUS toxicity (Ju et al., 2011). The yeast homologs of human proteins FBXW7 and EIF4A1 were also identified as suppressors. When tested in human cell lines with ALS-mutant FUS, both FBXW7 and EIF4A1 ameliorated FUS-induced toxicity (Sun et al., 2011).

Yeast two-hybrid screens also continue to be powerful methods to identify and evaluate interacting human proteins. A two-hybrid screen in yeast revealed that FUS interacts with the arginine-methylating protein PRMT1 (Yamaguchi and Kitajo, 2012). Methylation of FUS by PRMTs has subsequently been shown to be critical to mediating FUS subcellular localization (Tradewell et al., 2012; Yamaguchi and Kitajo, 2012).

Genetic screening in the TDP-43 yeast model has led to major ALS discoveries. A yeast deletion library was used to identify the DBR1 gene as a modifier of TDP-43 toxicity (Armakola et al., 2012; Figley and Gitler, 2013). Deletion of yeast DBR1 suppressed TDP-43-induced toxicity. This observation was recapitulated in cell lines and primary neurons, where inhibiting human Dbr1 similarly reduced TDP-43 toxicity. Dbr1 linearizes circular RNA resulting from splicing of pre-mRNA. Accumulation of circular RNA sequesters TDP-43, reducing its accumulation into more toxic aggregates, thus providing a conceptual therapeutic strategy.

Screening of a yeast over-expression library in conjunction with TDP-43 cytotoxicity led to the discovery of major genetic risk factors for ALS (Daigle et al., 2013). Expression of yeast PBP1 was found to specifically enhance TDP-43 cytotoxicity (it did not enhance the toxicity of other neurodegenerative-related proteins). The Pbp1 protein is homologous to human ataxin 2 (ATXN2), which itself causes spinocerebellar ataxia when its polyglutamine tract is expanded from a typical length of ~22 to >34. Immunohistochemical analysis of ALS patient spinal cord neurons revealed ATXN2 mislocalization. Sequencing of the ATXN2 gene in a large patient cohort revealed that intermediate polyglutamine expansion (~27–33) was a risk factor in ~5% of ALS cases. In subsequent studies, when ATXN2 levels were lowered in the central nervous system of a TDP-43-disease mouse model, survival was extended (Becker et al., 2017).

In addition to prion-like domains, many ALS-associated aggregation-prone proteins have RNA recognition motifs (RRMs). To determine if other RRM-containing proteins could be linked to disease, a yeast screen was performed with human RRM proteins (Couthouis et al., 2011). The aggregation and toxicity in yeast of 133 candidate proteins were evaluated, and then “hits” were further refined by bioinformatic sequence analysis of potential prion-like domains. TAF15 was found to aggregate, cause toxicity and have a prion-like domain that ranked highly according to prion-prediction algorithms. TAF15 was sequenced in ALS patients and several mutations were specific to patients and absent in control populations (Couthouis et al., 2011). Also, TAF15 was found to accumulate in the cytoplasm of spinal cord neurons of patients with sporadic ALS. Thus, screening in a yeast model enabled filtering a large pool of potential disease-associated proteins into the best candidates for more thorough analysis, which led to the discovery of a new ALS-linked protein.

### Dissecting the Roles of Disaggregases and Chaperones in Suppressing Protein-Aggregate-Induced Cytotoxicity

#### Hsp104

Work in the lab of James Shorter has identified yeast Hsp104 as having great therapeutic potential against both FUS and TDP-43, as well as other aggregation-prone proteins. Hsp104 is an ATP-dependent hexametric chaperone that disaggregates native prion amyloid in yeast. Its function in yeast prion propagation is critical because it breaks prion amyloid into smaller pieces that get passed to progeny cells (Wegrzyn et al., 2001; Helsen and Glover, 2012). This breaking of amyloid could have therapeutic potential in cases where long-lived cells must rid themselves of terminal protein aggregates. However, mammalian cells mysteriously do not code an Hsp104 homolog, despite no observed problems resulting from Hsp104 ectopic expression in mammalian cell models. In yeast models, Hsp104 has little effect on the aggregation and toxicity of ALS-linked proteins (Jackrel and Shorter, 2015). However, engineered variants of Hsp104 are capable of solubilizing FUS and TDP-43 aggregates and causing a concomitant reduction in cytotoxicity in yeast models (Jackrel et al., 2014; Tariq et al., 2018). This illustrates a case in which the yeast model system can be used to optimize new molecular technology for disaggregating amyloid-like inclusions.

An additional, subtler point emerges from these experiments. As Hsp104 is critical in disaggregating native yeast prions, the fact that it can also disaggregate ectopically expressed human proteins suggests that the aggregation of these proteins may be governed by prion-like biophysics.

#### Hsp40 Chaperones: Sis1 and DNAJB1

The yeast Hsp40 chaperone Sis1 interacts specifically with the amyloid forms of several yeast prions (Sondheimer et al., 2001; Higurashi et al., 2008). Work in the lab of Susan Liebman asked how Sis1 might similarly act upon aggregates formed by ALS-linked proteins. When over-expressed, the Sis1 suppresses proteotoxicity caused by both TDP-43 and FUS (Park et al., 2017, 2018). Importantly, this observation translated to mammalian cell models and primary cortical neurons, where increased expression of the human ortholog of Sis1, DNAJB1, could ameliorate TDP-43- and FUS-mediated toxicity (Park et al., 2017). This indicates DNAJB1 could be a therapeutic target of ALS subtypes. Human DNAJB1 can also alter yeast prions when expressed ectopically (Stein et al., 2014), thus yeast strains that are optimized for studying prions can also be used to study human chaperones linked to disease.

### Cross Seeding With Heterologous Amyloid

The nucleation of nearly all yeast prion proteins into self-propagating aggregates is influenced by the endogenous yeast prion [PIN^+^] (also known as [RNQ^+^]). The [PIN^+^] prion forms cytoplasmic amyloid that can nucleate the aggregation of heterologous proteins, particularly amyloid-forming prion proteins and polyglutamine expansions. Park and coworkers found that [PIN^+^] slightly enhances TDP-43 and FUS toxicity in yeast models (Park et al., 2017, 2018). These observations are consistent with TDP-43 and FUS having prion-like properties because it is hypothesized that amyloid structures have some potential to cross-template similar proteins into amyloid conformations. However, there was no evidence that FUS and TDP-43 strongly co-aggregated with the [PIN+] prion. Also, FUS and TDP-43 aggregates were not as resistant as prion proteins to detergent, which would suggest they are not in prion-like amyloid conformations.

The question remains about what conformations these ALS-linked prion-like proteins are forming when aggregated in diseased neurons. *In vitro*, the large aggregates formed by these proteins are described as “amyloid-like,” which indicates they appear to form fibrous solid aggregates with mild detergent-resistance and weak or no Thioflavin T reactivity (Johnson et al., 2008; Fushimi et al., 2011). Thioflavin T binding and fluorescence are frequently used to characterize amyloid, so in the absence of a strong Thioflavin T fluorescence, aggregates may be dismissed as non-amyloid. However, there is no molecular explanation that would require this to always be true. For example, recombinant FUS prion-like domain forms archetypal amyloid fibers with cross-beta structure, but are not Thioflavin T responsive (Murray et al., 2017). This indicates that the ALS-linked proteins may be forming a type of amyloid that does not react strongly with Thioflavin T.

## Limitations of Yeast Models

There are limitations to yeast models. What is true for yeast prion domains is not necessarily true for human prion-like domains, so conclusions must be made carefully. For example, it has been hypothesized that prion formation in yeast is naturally beneficial (True et al., 2004; Halfmann et al., 2012), which is not hypothesized for the human ALS proteins with prion-like domains. In fact, nearly all mammalian prion-like proteins are novel since the last common ancestor with yeast (An and Harrison, 2016). This means the similarity in amino-acid composition is a result of convergence, not because the precise properties of yeast prion domains have been conserved through evolutionary time. The differences and similarities between prion and prion-like domains is an ongoing topic of research.

An additional limitation of yeast models is different sets of resident interacting proteins compared to mammalian cells. This is especially important in regards to quality-control proteins like chaperons. Yeast chaperones are integral to propagation of endogenous yeast prions (Masison and Reidy, 2015), but their interactions with ectopically expressed human prion-like proteins is artificial, so may not be informative to human disease. However, as mentioned above, the absence of human protein-quality-control factors can be an advantage; pairing human chaperones with human disease proteins in yeast can be used to evaluate chaperone-mediated changes to aggregation and toxicity. Numerous examples of human chaperones reducing human disease protein gain-of-function toxicity in yeast have been published (De Graeve et al., 2013; Kumar et al., 2018; Park et al., 2018).

## Concluding Remarks

Yeast models have seen widespread adoption for modeling human neurodegenerative diseases like ALS because they are easily manipulated and recapitulate the major properties of more complex eukaryotic cells. Specifically, these yeast models offer insights into the precise relationship between gain-of-function toxicity and protein aggregation. While they do not recapitulate the numerous mechanisms by which neuronal networks may degenerate, yeast models have proven effective in discovering proteins linked to ALS and FTD. Yeast models of prion-like neurodegenerative disease-linked proteins offer a strategy to inexpensively inform subsequent experiments in higher model systems in the goal of ultimately developing therapeutics.

## Author Contributions

ZM and FS conceived the outline. ZM, SR, DY and FS wrote the article.

## Conflict of Interest Statement

The authors declare that the research was conducted in the absence of any commercial or financial relationships that could be construed as a potential conflict of interest.
